# Supplementary material for: Systems approach for congruence and selection of cancer models towards precision medicine
Source: PLoS Comput Biol. 2024 Jan 10;20(1):e1011754. doi: 10.1371/journal.pcbi.1011754 (PMC10805322; doi:10.1371/journal.pcbi.1011754)
Supplement: S1 Table — (DOCX) [file pcbi.1011754.s001.docx]

**S1 Table.** Summary of relevant publications.

|  | Evaluating the transcriptional fidelity of cancer models, *Genome Medicine*, April 2021[1] | | Global computational alignment of tumor and cell line transcriptional profiles , *Nature Communications,* 04 January 2021[2] | | CCLA: an accurate method and web server for cancer cell line authentication using gene expression profiles, *Briefings in Bioinformatics,* 08 June 2020[3] | | Matching cell lines with cancer type and subtype of origin via mutational, epigenomic, and transcriptomic patterns, *Science Advances*, 01 Jul 2020[4] | |  |
| --- | --- | --- | --- | --- | --- | --- | --- | --- | --- |
| Dataset | TCGA, CCLE, ICGC, etc. | | Treehouse, CCLE | | CCLE, GDSC, CHCC | | CCLE, TCGA | |  |
| Method  Evaluation | 1. Identify upregulated, downregulated, and invariant genes in each tumor type by template vector and Pearson correlation 2. Select the most discriminative gene pairs for each tumor type from the above identified genes 3. Train the random forest model using above selected gene pairs 4. Evaluate the cancer models on the 22 tumor types and 36 sub-types 5. Evaluate the similarity in cancer cell lines, xenografts, mouse models, and tumoroids | | Propose a method to perform an unsupervised global alignment of tumor and cell line gene expressions, allowing for direct comparisons of their transcriptional profiles.   1. Calculate the Pearson correlation between aligned tumor and cell line 2. Cell lines are classiﬁed by identifying the most frequently occurring tumor type within each cell line’s 25 highest correlated tumors 3. Show the information transformation between cell and tumor 4. Validate the method using known truth | | 1. Apply *single sample gene set enrichment analysis* to the reference set for obtaining the reference score matrix 2. Cluster the reference score matrix into 3 groups by t-SNE 3. Apply random forest using the above group label 4. Obtain the group label for the new sample, and calculate the Pearson correlation with the reference samples within that group 5. Use independent data source 6. Compare the distribution of expressed signature genes in the query samples and resulting reference | | 1. Construct the one vs. rest ridge regression model for each cancer type using TCGA methylation and gene expression data respectively 2. Select the cell lines which have high precision score in both models but reported from the other origin 3. Construct the one (origin) vs. one (suspected) model for the above selected cell lines 4. Verify the misclassification using other mutant data 5. Validate the misclassification using cancer type-specific drugs and specific mutation signatures 6. Use UV-linked signature 7 and sensitivity (IC_50_) for mutant target drugs to evaluate the 6 cell lines which are consistently reassigned to skin cancer 7. Subtype the cell lines and validate the results using breast cancer cell line subtyping labels 8. Perform association study using different set of cell lines on drug sensitivity and gene dependency screenings | |  |
| Pros | 1. The method is platform- and species - agnostic because of ranked-based design 2. Many cancer models are studied | | 1. New method to make the cell line and tumor data comparable | | 1. User-friendly web sever 2. Gene signatures for each cell line are carefully selected | | 1. Apply multi-omics data, especially drug sensitivity data 2. Classification model works well | |  |
| Cons | 1. Cannot measure absolute distance between cell line and tumor 2. The comparability between models and tumors is not considered | | 1. The classification method does not provide enough information | | 1. Not consider the tumor | | 1. Cannot measure absolute distance between cell line and tumor | |  |
|  | | Evaluating cell lines as tumor models by comparison of genomic profiles, *Nature Communications*, 09 July 2013[5] | | Integrated analyses of murine breast cancer models reveal critical parallels with human disease, *Nature Communications*, 22 July 2019[6] | | Comprehensive transcriptomic analysis of cell lines as models of primary tumors across 22 tumor types, *Nature Communications,* 08 August 2019[7] | | Evaluating cell lines as models for metastatic breast cancer through integrative analysis of genomic data, *Nature Communications*, 15 May 2019[8] | |
| Dataset | | CCLE, TCGA | | Lab data | | TCGA, CCLE | | MET500, CCLE | |
| Method | | Suitability Score:  S = A + B – 2 X C – D/7   1. A: Correlation with mean CNA of HGSOC tumors 2. B: 1 or 0, TP53 mutation 3. C: 1 or 0, hypermutated 4. D: number of genes mutated among 7 ’non-HGSOC’ genes | | 1. Filter the resulting genes based on human data and cluster on gene expression 2. Identify genes highly altered in human, and analyzing CNV in mouse | | 1. Correlation analysis (remove the tumor purity genes) and gene set enrichment analysis 2. *Nearest Template Prediction* for subtype prediction of cell lines | | 1. Compare genomic proﬁles (Genes highly mutated in metastatic breast cancer & differentially mutated between metastatic and primary breast cancer) 2. Spearman correlation across 1,000 most-varied genes | |
| Pros | | Design a new score including the important factors | | The evaluation of mouse model is based on human information | | 1. Remove the tumor purity genes to make cell lines and tumors more comparable 2. Generate subtype templates using specific genes for NTP | | 1. Focus on metastasis instead of primary tumor 2. Important gene selection part is thought-provoking | |
| Cons | | The score is specific for this one case, and how this score designed (the weights) is not fully illustrated | | The correlation between human and mouse model is not fully compared | | Cannot measure absolute distance between cell line and tumor | | Cannot measure absolute distance between cell line and tumor | |

|  | Analysis of transcriptomic similarity between osteosarcoma cell lines and primary tumors, *Oncology,* 23 Jul 2020[9] | Assessing alveolar rhabdomyosarcoma cell lines as tumor models by comparison of mRNA expression profiles, *Gene*, 15 November 2020[10] | Investigating the utility of human melanoma cell lines as tumour models, *Oncotarget*, 7 Feb 2017[11] |
| --- | --- | --- | --- |
| Dataset | TCGA, CCLE | CCLE, GEO | TCGA, GEO |
| Method | 1. Calculate spearman correlation using 5,000 top genes (by IQR) from primary tumor 2. Differential expression analysis for tumor versus cell lines with purity score and sequencing platform as covariate 3. Gene ontology enrichment analysis based on DEA results | | 1. PCA on cell lines and tumors by top 5,000 genes 2. DEA on cell lines versus tumors 3. Calculate the Pearson association between RNA-seq of cell lines and single cells 4. Subtype the cell lines by clustering using 2 gene sets identified in tumors 5. Detect the UV-induced mutational signatures 6. Prepare a panel for selection based on average properties and genetic events from tumor study |
| Pros | 1. It is interesting to use DEA to identify genes that are differentially expressed between cell lines and primary tumors | | 1. Use PCA to evaluate the performance of batch correction method 2. A relatively complete analysis 3. Use important signatures for validation 4. Use a panel to summarize the results |
| Cons | 1. Cannot measure absolute distance between cell line and tumor 2. Cannot perform the cell line selection | | 1. The correlation calculation is too simple 2. The resulting panel cannot directly provide a selection suggestion |

**Reference**

1. Peng D, Gleyzer R, Tai W-H, Kumar P, Bian Q, Isaacs B, et al. Evaluating the transcriptional fidelity of cancer models. Genome Med. 2021;13: 73.

2. Warren A, Chen Y, Jones A, Shibue T, Hahn WC, Boehm JS, et al. Global computational alignment of tumor and cell line transcriptional profiles. Nat Commun. 2021;12: 22.

3. Zhang Q, Luo M, Liu C-J, Guo A-Y. CCLA: an accurate method and web server for cancer cell line authentication using gene expression profiles. Brief Bioinform. 2021;22. doi:10.1093/bib/bbaa093

4. Salvadores M, Fuster-Tormo F, Supek F. Matching cell lines with cancer type and subtype of origin via mutational, epigenomic, and transcriptomic patterns. Sci Adv. 2020;6. doi:10.1126/sciadv.aba1862

5. Domcke S, Sinha R, Levine DA, Sander C, Schultz N. Evaluating cell lines as tumour models by comparison of genomic profiles. Nat Commun. 2013;4: 2126.

6. Rennhack JP, To B, Swiatnicki M, Dulak C, Ogrodzinski MP, Zhang Y, et al. Integrated analyses of murine breast cancer models reveal critical parallels with human disease. Nat Commun. 2019;10: 3261.

7. Yu K, Chen B, Aran D, Charalel J, Yau C, Wolf DM, et al. Comprehensive transcriptomic analysis of cell lines as models of primary tumors across 22 tumor types. Nat Commun. 2019;10: 3574.

8. Liu K, Newbury PA, Glicksberg BS, Zeng WZD, Paithankar S, Andrechek ER, et al. Evaluating cell lines as models for metastatic breast cancer through integrative analysis of genomic data. Nat Commun. 2019;10: 2138.

9. Batchu S, Gold JL. Analysis of transcriptomic similarity between osteosarcoma cell lines and primary tumors. Oncology. 2020;98: 814–816.

10. Batchu S, Kellish AS, Hakim AA. Assessing alveolar rhabdomyosarcoma cell lines as tumor models by comparison of mRNA expression profiles. Gene. 2020;760: 145025.

11. Vincent KM, Postovit L-M. Investigating the utility of human melanoma cell lines as tumour models. Oncotarget. 2017;8: 10498–10509.
