# Supplementary material for: Systems approach for congruence and selection of cancer models towards precision medicine
Source: PLoS Comput Biol. 2024 Jan 10;20(1):e1011754. doi: 10.1371/journal.pcbi.1011754 (PMC10805322; doi:10.1371/journal.pcbi.1011754)
Supplement: S3 Table — (DOCX) [file pcbi.1011754.s003.docx]

**S3 Table.** Summary table of the 38 candidate BC cell lines.

|  | Projected Position | $P_{SDA}^{ILC}$ | ${DS}_{SDA}^{ILC}$ | Classification |
| --- | --- | --- | --- | --- |
| SUM44PE | -0.977 | 0.987 | 0.024 | ILC |
| DU4475 | -1.036 | 0.992 | 0.188 | ILC |
| UACC3133 | -1.132 | 0.996 | 0.452 | ILC |
| CAMA1 | -0.771 | 0.929 | 0.541 | ILC |
| HCC2218 | -0.666 | 0.845 | 0.828 | ILC |
| ZR7530 | -0.604 | 0.764 | 0.999 | ILC |
| WCRC25 | -1.333 | 0.999 | 1.002 | ILC |
| MDAMB453 | -0.549 | 0.670 | 1.150 | ILC |
| MDAMB134VI | -0.488 | 0.548 | 1.318 | ILC |
| BCK4 | -1.544 | 1.000 | 1.583 | ILC |
| OCUBM | -0.386 | 0.339 | 1.597 | No ILC |
| IPH926 | -1.551 | 1.000 | 1.601 | ILC |
| UACC893 | -0.361 | 0.293 | 1.667 | No ILC |
| MDAMB175VII | -0.326 | 0.236 | 1.762 | No ILC |
| HCC2185 | -1.657 | 1.000 | 1.892 | ILC |
| T47D | -0.264 | 0.155 | 1.934 | No ILC |
| MPE600 | -0.235 | 0.125 | 2.013 | No ILC |
| HCC1428 | -0.222 | 0.114 | 2.050 | No ILC |
| CAL148 | -0.200 | 0.097 | 2.108 | No ILC |
| SUM185PE | -0.193 | 0.091 | 2.129 | No ILC |
| HCC1419 | -0.179 | 0.082 | 2.166 | No ILC |
| AU565 | -1.844 | 1.000 | 2.405 | ILC |
| SUM52PE | -0.044 | 0.028 | 2.538 | No ILC |
| SKBR3 | -1.92 | 1.000 | 2.615 | ILC |
| BT483 | 0.083 | 0.01 | 2.885 | No ILC |
| MDAMB415 | 0.088 | 0.009 | 2.899 | No ILC |
| MM330 | 0.103 | 0.008 | 2.942 | No ILC |
| ZR751 | -2.050 | 1.000 | 2.970 | No ILC |
| UACC812 | -2.057 | 1.000 | 2.991 | No ILC |
| HCC1500 | 0.254 | 0.002 | 3.356 | No ILC |
| MFM223 | 0.334 | 0.001 | 3.577 | No ILC |
| MDAMB361 | 0.479 | 0.000 | 3.974 | No ILC |
| HCC202 | 0.512 | 0.000 | 4.066 | No ILC |
| KPL1 | 0.543 | 0.000 | 4.150 | No ILC |
| EFM19 | 0.649 | 0.000 | 4.439 | No ILC |
| MCF7 | 0.745 | 0.000 | 4.705 | No ILC |
| EFM192A | 0.800 | 0.000 | 4.856 | No ILC |
| BT474 | 1.627 | 0.000 | 7.127 | No ILC |
