# Supplementary material for: Systems approach for congruence and selection of cancer models towards precision medicine
Source: PLoS Comput Biol. 2024 Jan 10;20(1):e1011754. doi: 10.1371/journal.pcbi.1011754 (PMC10805322; doi:10.1371/journal.pcbi.1011754)
Supplement: S4 Table — (DOCX) [file pcbi.1011754.s004.docx]

**S4 Table.** Summary table of the pathway specific analysis ($DS_{Path}$) for 9 unbiased-selected + 1 manually-included cell lines and 14 unbiased-selected + 1 manually-included pathways.

|  | SUM44PE | DU4475 | UACC3133 | CAMA1 | HCC2218 | WCRC25 | BCK4 | IPH926 | HCC2185 | MDAMB134VI |
| --- | --- | --- | --- | --- | --- | --- | --- | --- | --- | --- |
| KEGG_PPAR_SIGNALING_PATHWAY | 0.405 | 0.572 | 0.42 | 0.493 | 0.349 | 0.358 | 0.72 | 0.615 | 0.395 | 0.418 |
| HALLMARK_TNFA_SIGNALING_VIA_NFKB | 0.589 | 0.984 | 0.877 | 0.369 | 0.644 | 0.635 | 0.875 | 0.599 | 0.932 | 0.53 |
| HALLMARK_KRAS_SIGNALING_DN | 0.730 | 0.667 | 0.558 | 0.456 | 0.572 | 0.645 | 0.813 | 0.553 | 0.863 | 0.636 |
| HALLMARK_GLYCOLYSIS | 0.958 | 0.970 | 0.816 | 0.591 | 0.800 | 0.990 | 1.009 | 0.791 | 0.861 | 0.562 |
| KEGG_STARCH_AND_SUCROSE_METABOLISM | 0.868 | 0.581 | 0.620 | 0.425 | 0.623 | 0.915 | 0.785 | 1.284 | 0.669 | 0.464 |
| HALLMARK_SPERMATOGENESIS | 0.430 | 0.875 | 0.513 | 0.465 | 0.904 | 0.535 | 0.507 | 0.624 | 0.438 | 0.64 |
| HALLMARK_MYC_TARGETS_V1 | 0.525 | 0.849 | 0.497 | 0.488 | 0.991 | 0.456 | 0.529 | 0.551 | 0.556 | 0.566 |
| KEGG_CELL_CYCLE | 0.572 | 0.755 | 0.363 | 0.579 | 0.992 | 0.548 | 0.45 | 0.655 | 0.582 | 0.627 |
| KEGG_METABOLISM_OF_XENOBIOTICS_BY_CYTOCHROME_P450 | 0.822 | 0.543 | 1.050 | 0.528 | 0.508 | 0.837 | 0.911 | 0.972 | 0.638 | 0.49 |
| KEGG_RETINOL_METABOLISM | 1.051 | 0.540 | 0.806 | 0.453 | 0.541 | 0.981 | 0.932 | 1.062 | 0.997 | 0.452 |
| HALLMARK_MTORC1_SIGNALING | 0.88 | 1.040 | 0.689 | 0.619 | 0.778 | 0.768 | 0.898 | 0.951 | 0.715 | 0.958 |
| KEGG_DRUG_METABOLISM_CYTOCHROME_P450 | 0.768 | 0.499 | 1.035 | 0.498 | 0.482 | 0.823 | 0.832 | 0.991 | 0.611 | 0.439 |
| HALLMARK_G2M_CHECKPOINT | 0.478 | 0.609 | 0.458 | 0.525 | 1.138 | 0.451 | 0.376 | 0.391 | 0.425 | 0.531 |
| HALLMARK_E2F_TARGETS | 0.569 | 0.799 | 0.629 | 0.576 | 1.156 | 0.389 | 0.440 | 0.520 | 0.470 | 0.632 |
| AVERAGE OF 14 PATHWAYS | 0.689 | 0.734 | 0.667 | 0.505 | 0.748 | 0.666 | 0.72 | 0.754 | 0.654 | 0.567 |
