# Supplementary material for: Systems approach for congruence and selection of cancer models towards precision medicine
Source: PLoS Comput Biol. 2024 Jan 10;20(1):e1011754. doi: 10.1371/journal.pcbi.1011754 (PMC10805322; doi:10.1371/journal.pcbi.1011754)
Supplement: S5 Table — (DOCX) [file pcbi.1011754.s005.docx]

**S5 Table.** Confusion matrix of genome-wide predication for HER2+, ER+/PR+, and TNBC prediction.

|  | | Prediction | | | |
| --- | --- | --- | --- | --- | --- |
|  |  | ER+/PR+/HER2- | HER2+ | TNBC | None |
| Annotation | ER+/PR+/HER2- | 5 | 0 | 0 | 1 |
|  | HER2+ | 1 (UACC812) | 8 | 0 | 3 |
|  | TNBC | 1 (HCC1500) | 0 | 12 | 12 |
