# Supplementary material for: Systems approach for congruence and selection of cancer models towards precision medicine
Source: PLoS Comput Biol. 2024 Jan 10;20(1):e1011754. doi: 10.1371/journal.pcbi.1011754 (PMC10805322; doi:10.1371/journal.pcbi.1011754)
Supplement: S6 Table — (DOCX) [file pcbi.1011754.s006.docx]

**S6 Table.** Summary table of the 11 PDO and 136 PDX BC models.

| Patient ID | Specimen ID | Sample ID | Projected Position | $P_{SDA}^{ILC}$ | ${DS}_{SDA}^{ILC}$ | Classification |
| --- | --- | --- | --- | --- | --- | --- |
| 171881 | 019-R | APW-DS2 | 1.072 | 0.998 | 0.117 | ILC |
| 171881 | 019-R | APYF68 | 0.915 | 0.987 | 0.562 | ILC |
| 171881 | 019-R | APWG05 | 0.706 | 0.898 | 1.154 | ILC |
| 171881 | 019-R | V1-organoid | 0.501 | 0.508 | 1.733 | ILC |
| 171881 | 019-R | APWG05PF7 | 0.432 | 0.335 | 1.928 | No ILC |
| 337426 | 197-R | AL-F5Y_AL-A80 | 0.036 | 0.008 | 3.05 | No ILC |
| 755229 | 096-R | AL-VNC_AL-C53_AL-J67 | 0.004 | 0.006 | 3.141 | No ILC |
| 755229 | 096-R | AL-VNC_AL-C53_AL-J67_AL-Q60 | -0.026 | 0.004 | 3.227 | No ILC |
| 337426 | 197-R | AL-F5Y | -0.039 | 0.004 | 3.261 | No ILC |
| 397859 | 316-R | P0POOL_OT-Q25 | -0.044 | 0.003 | 3.275 | No ILC |
| 337426 | 197-R | AL-F5W_AL-A70 | -0.067 | 0.003 | 3.34 | No ILC |
| 755229 | 096-R | AL-VNC_AL-C54_AL-Q07 | -0.068 | 0.003 | 3.343 | No ILC |
| 397859 | 316-R | P0POOL_OT-Q25_RG-NP9 | -0.116 | 0.002 | 3.48 | No ILC |
| 337426 | 197-R | AL-F5Y_AL-A81_AL-C56_AL-E24_AL-F39 | -0.13 | 0.001 | 3.521 | No ILC |
| 171881 | 019-R | APVG40_RG-G15 | -0.145 | 0.001 | 3.562 | No ILC |
| 755229 | 096-R | AL-VNC_AL-C55 | -0.186 | 0.001 | 3.68 | No ILC |
| 755229 | 096-R | AL-VNC | -0.191 | 0.001 | 3.691 | No ILC |
| 397859 | 316-R | P0POOL_OT-Q23N59 | -0.223 | 0.001 | 3.783 | No ILC |
| 755229 | 096-R | V1-organoid | -0.25 | 0 | 3.861 | No ILC |
| 337426 | 197-R | V2-organoid | -0.274 | 0 | 3.929 | No ILC |
| 913291 | 066-R | V1-organoid | -0.316 | 0 | 4.047 | No ILC |
| 397859 | 316-R | P0POOL_OT-Q23 | -0.331 | 0 | 4.09 | No ILC |
| 913291 | 066-R | UJH | -0.338 | 0 | 4.109 | No ILC |
| 913291 | 066-R | UJHG08 | -0.391 | 0 | 4.259 | No ILC |
| 913291 | 066-R | UJHG07K01 | -0.393 | 0 | 4.266 | No ILC |
| 913291 | 066-R | UJHG08J25 | -0.412 | 0 | 4.319 | No ILC |
| 397859 | 316-R | P0POOL_OT-Q23N60KY7W19 | -0.517 | 0 | 4.617 | No ILC |
| 913291 | 066-R | UJF | -0.631 | 0 | 4.939 | No ILC |
| 913291 | 066-R | UJHG08J26_AL-KX9 | -0.728 | 0 | 5.214 | No ILC |
