# Supplementary figures and images for: Systems approach for congruence and selection of cancer models towards precision medicine
Source: PLoS Comput Biol. 2024 Jan 10;20(1):e1011754. doi: 10.1371/journal.pcbi.1011754 (PMC10805322; doi:10.1371/journal.pcbi.1011754)

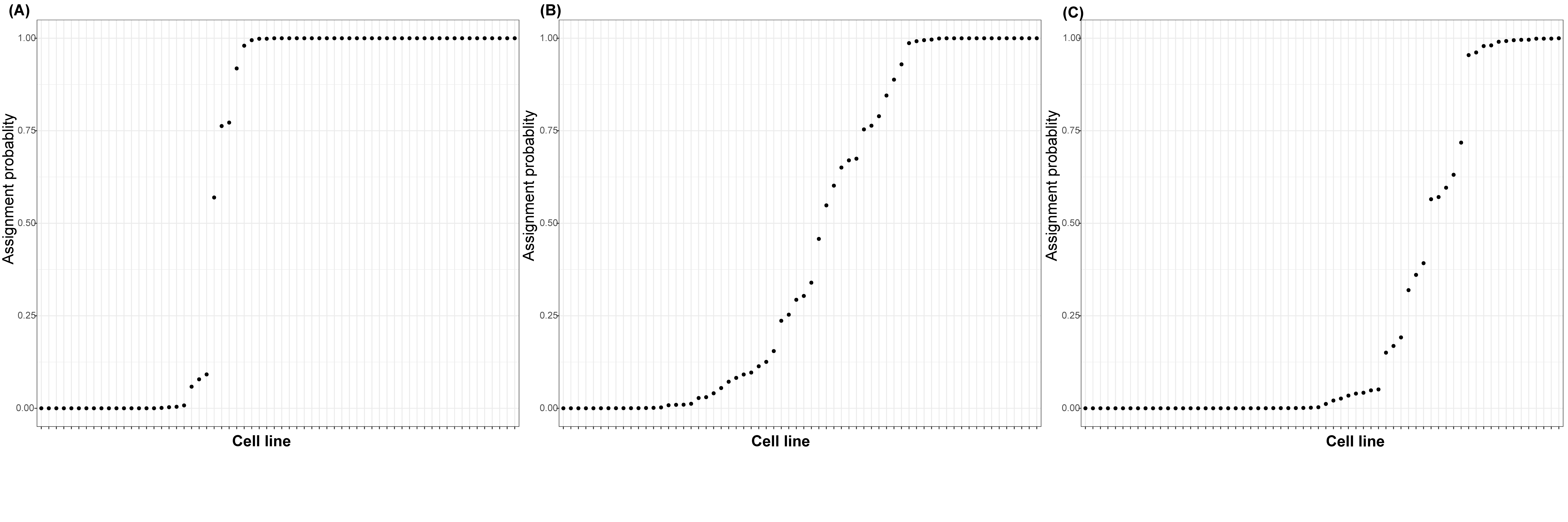

Supplement: S1 Fig — (A) LDA; (B) SDA; (C) RSLDA. (TIF) [file pcbi.1011754.s007.tif]

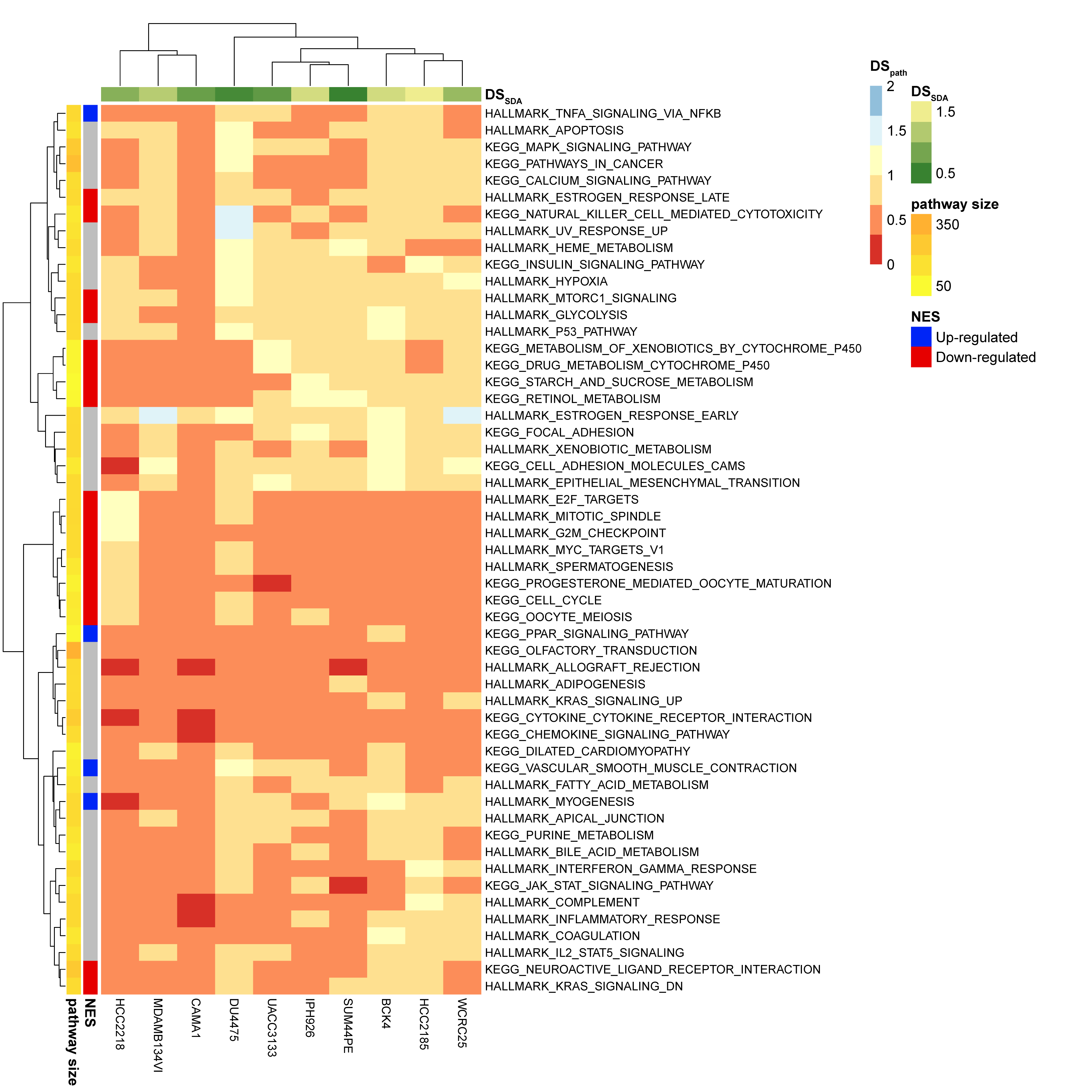

Supplement: S2 Fig — The genome-wide SDA projected deviance score (DSSDA) is shown on the top sidebar and the pathway size and normalized enrichment score (NES) are on the left. (TIF) [file pcbi.1011754.s008.tif]

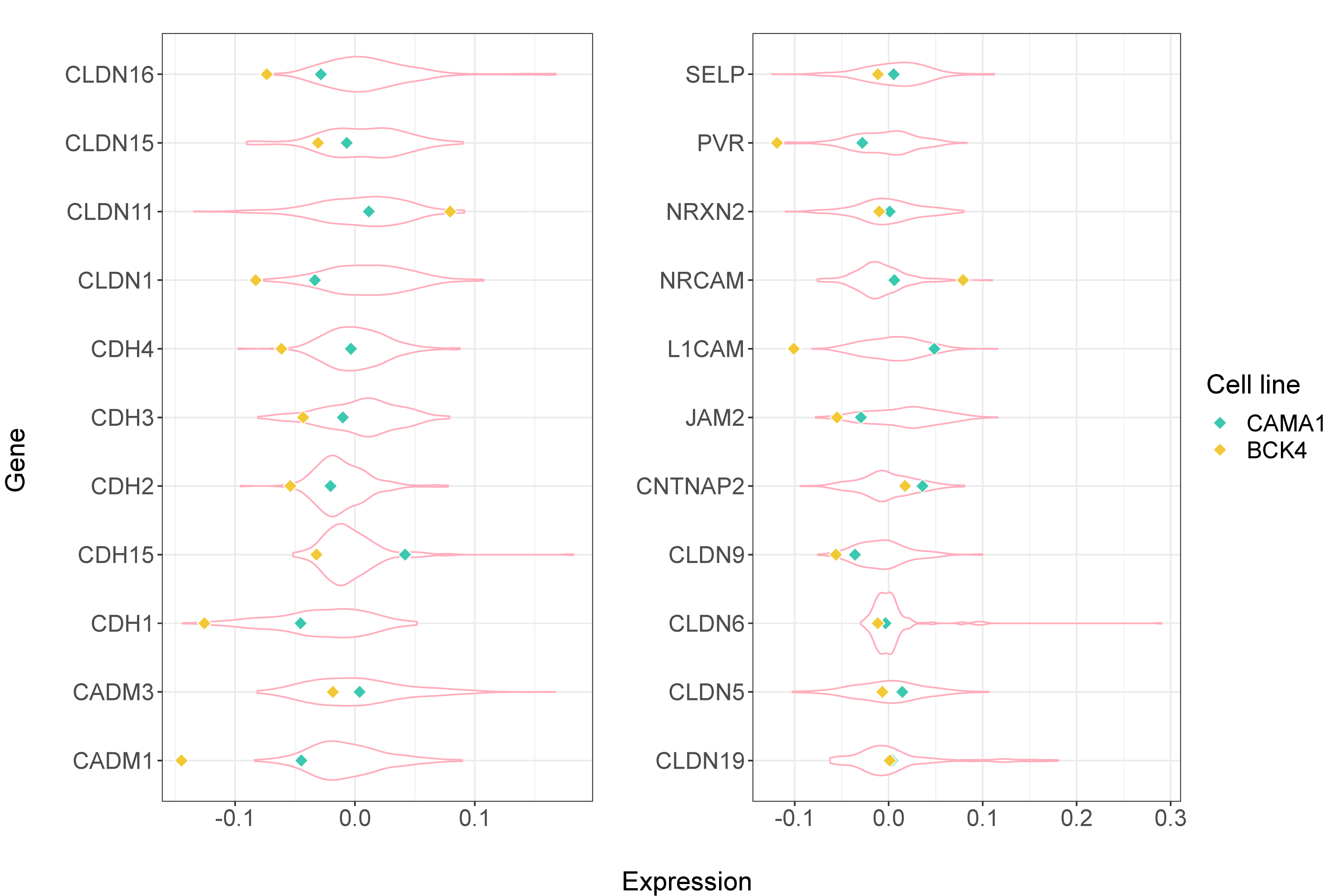

Supplement: S3 Fig — (TIF) [file pcbi.1011754.s009.tif]

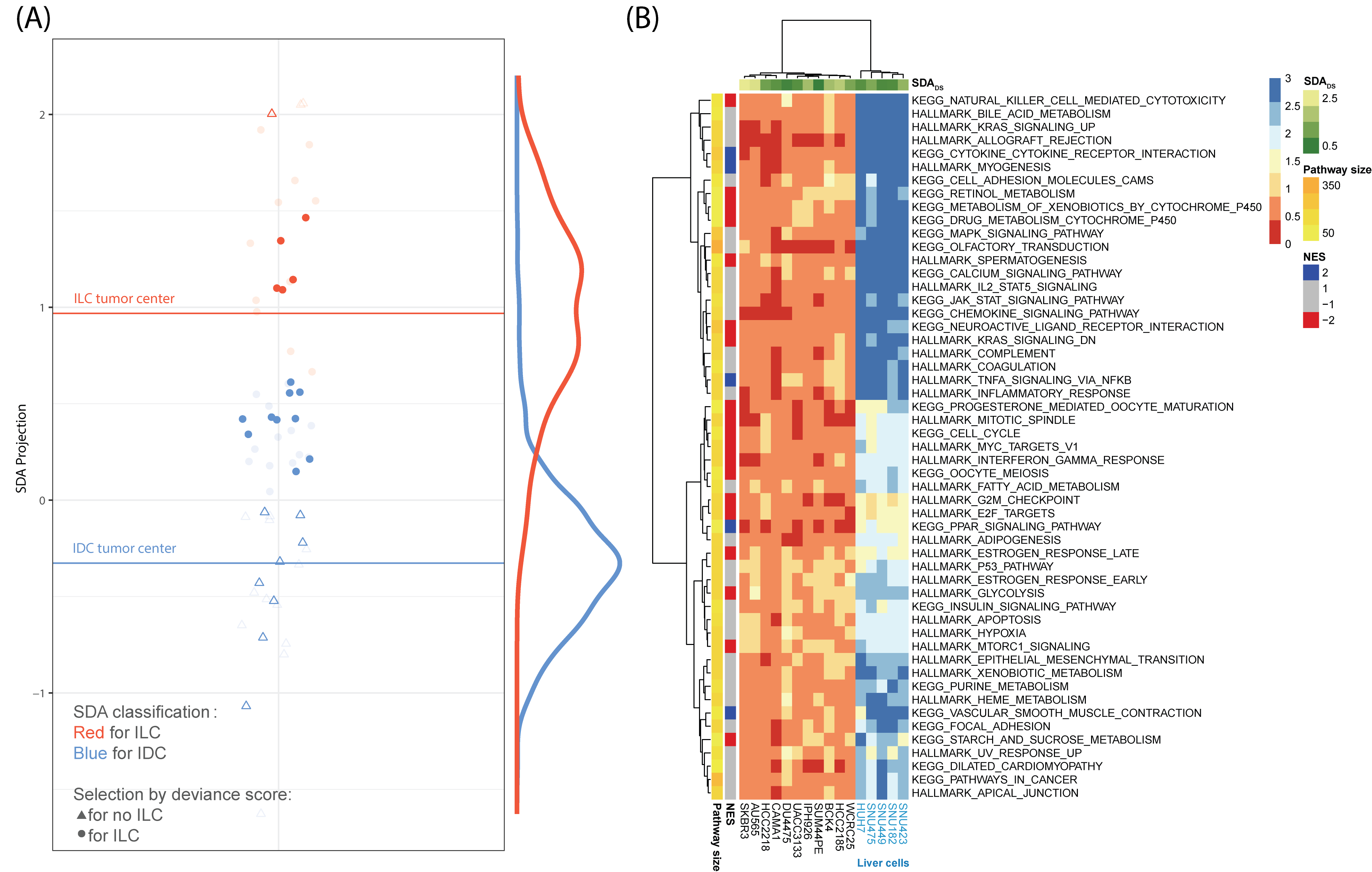

Supplement: S4 Fig — (A) Genome-wide preselection identifies 5 out of 24 liver cell lines as ILC. (B) Pathway-based heatmap reveals that these 5 preselected cell lines significantly diverge from the ILC tumor center in terms of differentially expressed genes and associated pathways. (TIF) [file pcbi.1011754.s010.tif]

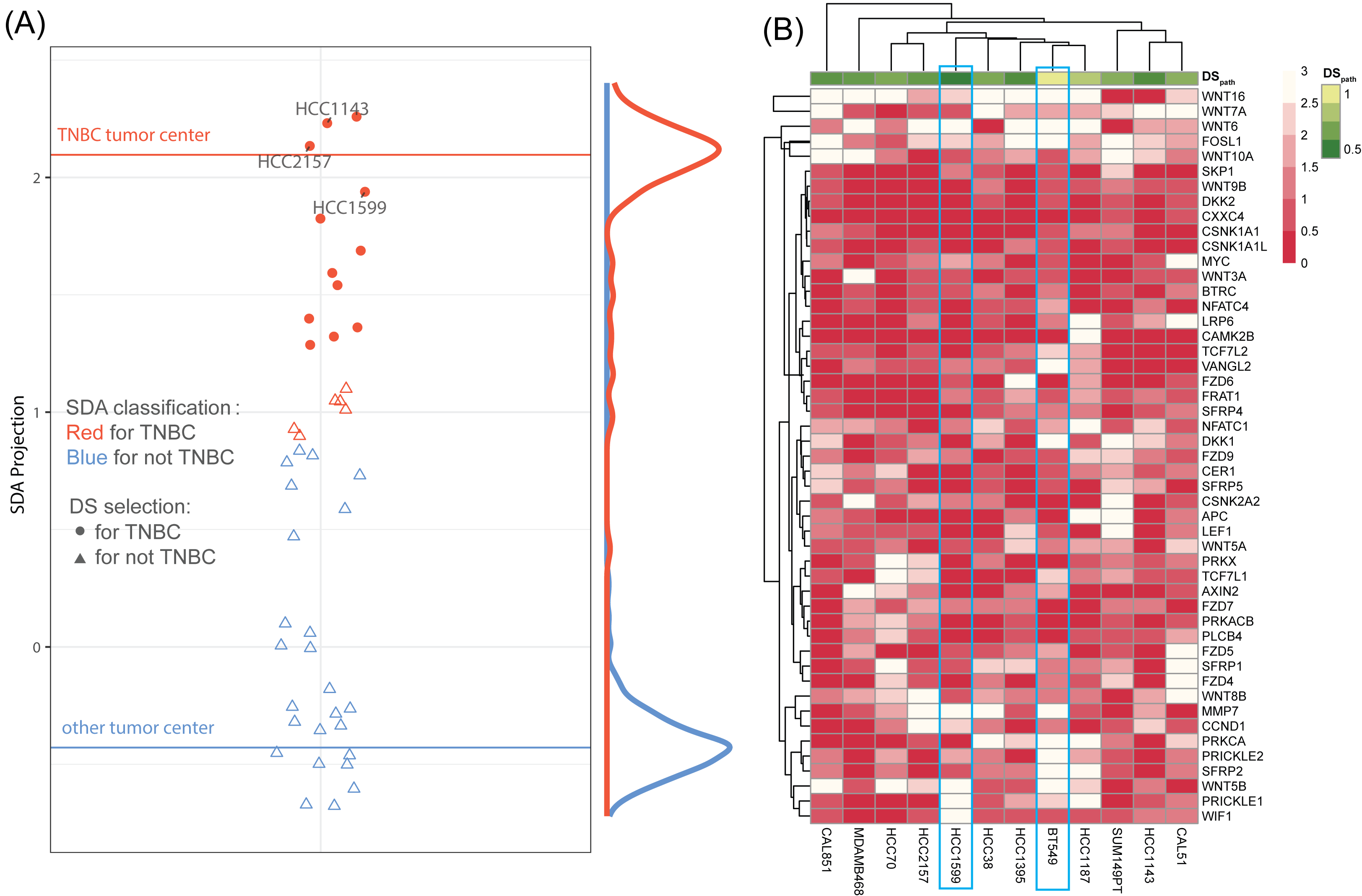

Supplement: S5 Fig — (A) SDA projected scatterplot shows the position of each candidate cell line and 12 cell lines were selected for down-stream analysis. (B) heatmap for the comparison of cell lines in KEGG WNT signaling pathway (BT549 and HCC1599 highlighted). (TIF) [file pcbi.1011754.s011.tif]

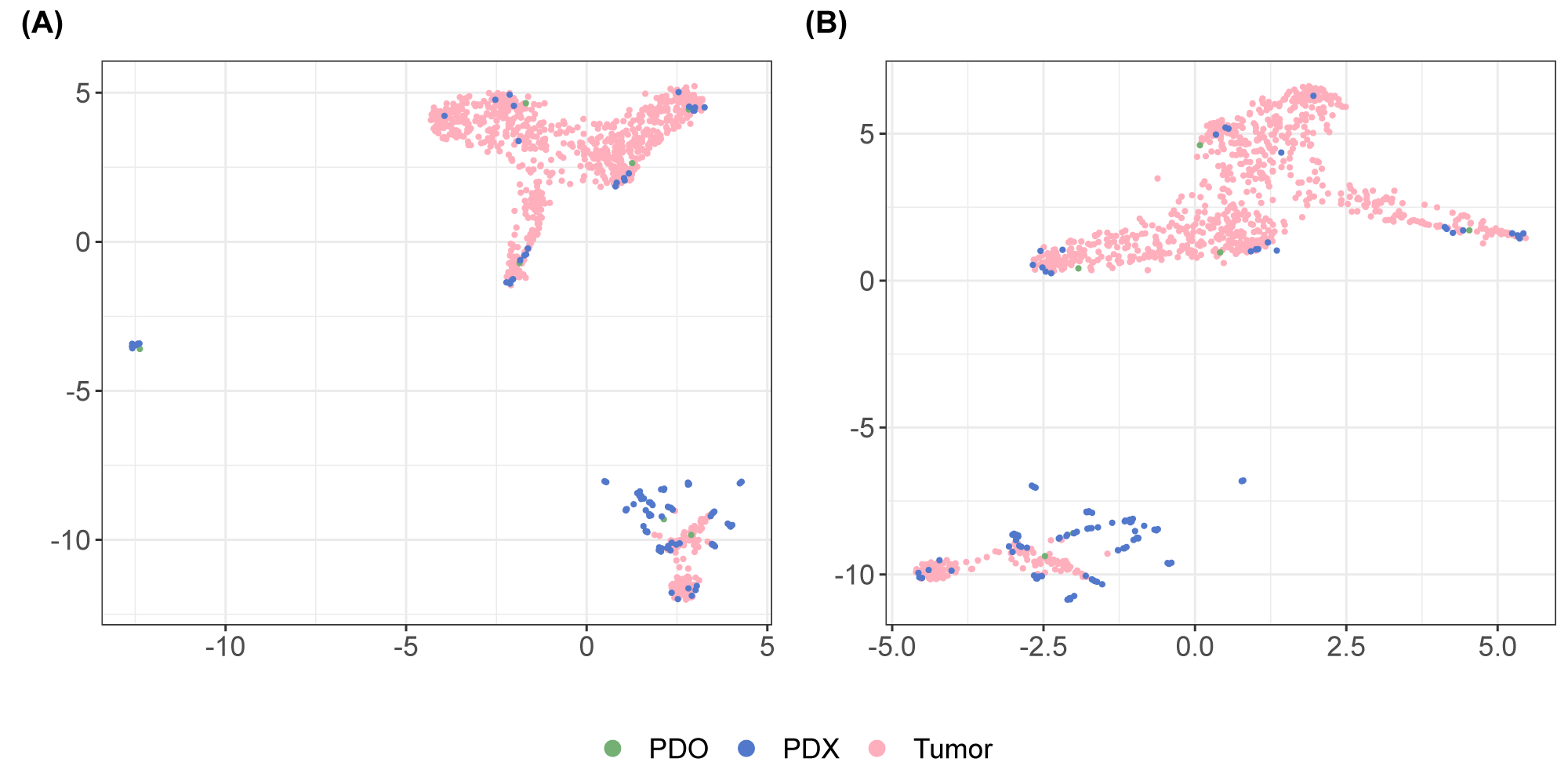

Supplement: S6 Fig — (A) Three distinct clusters were observed. The small cluster on the left consists of a seemingly rare breast cancer subtype, the upper-right cluster includes mostly non-basal samples, and the lower-right cluster includes mostly basal samples. (B) UMAP is redrawn when the small cluster in (A) is removed. (TIF) [file pcbi.1011754.s012.tif]

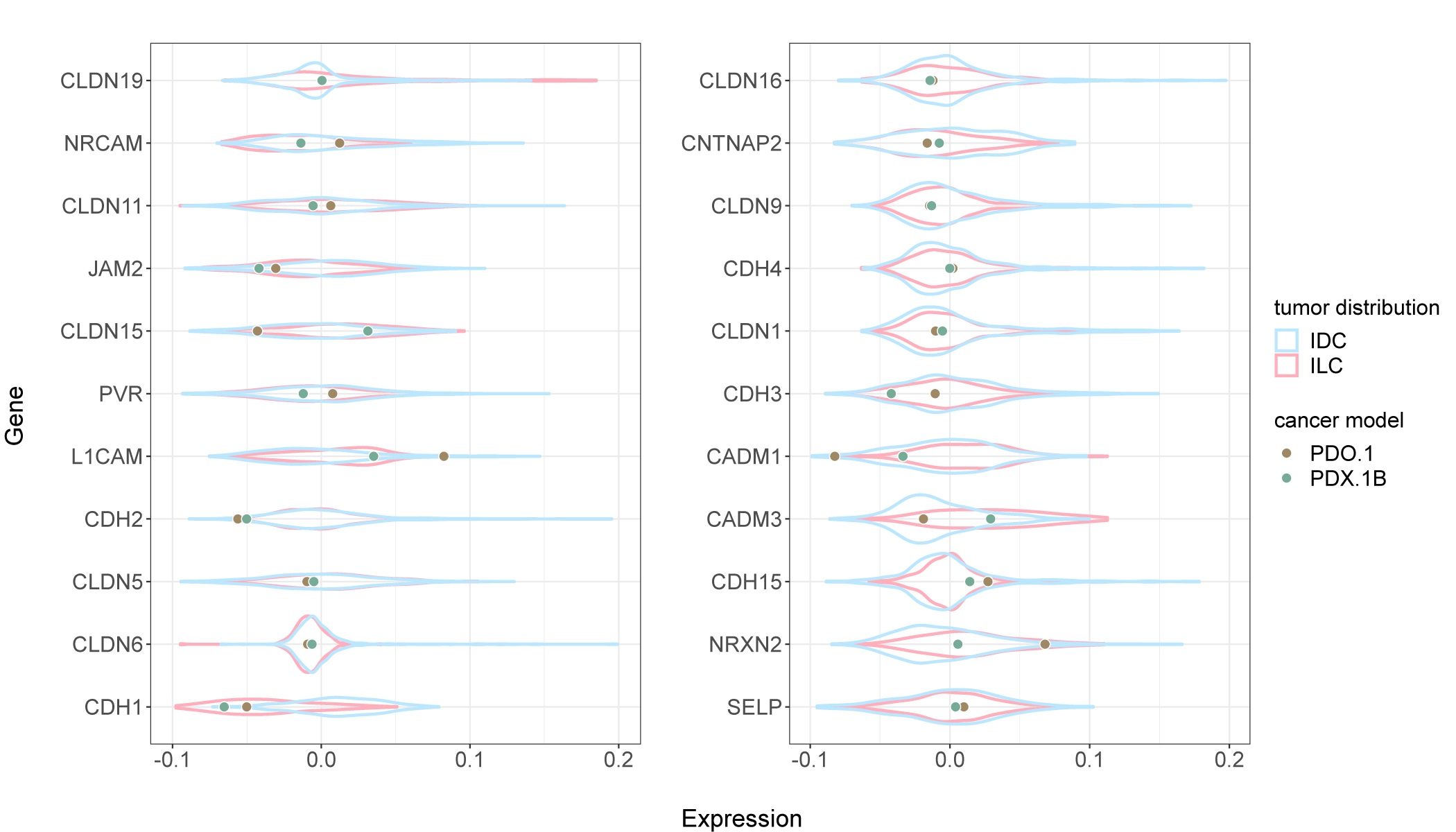

Supplement: S7 Fig — (TIF) [file pcbi.1011754.s013.tif]

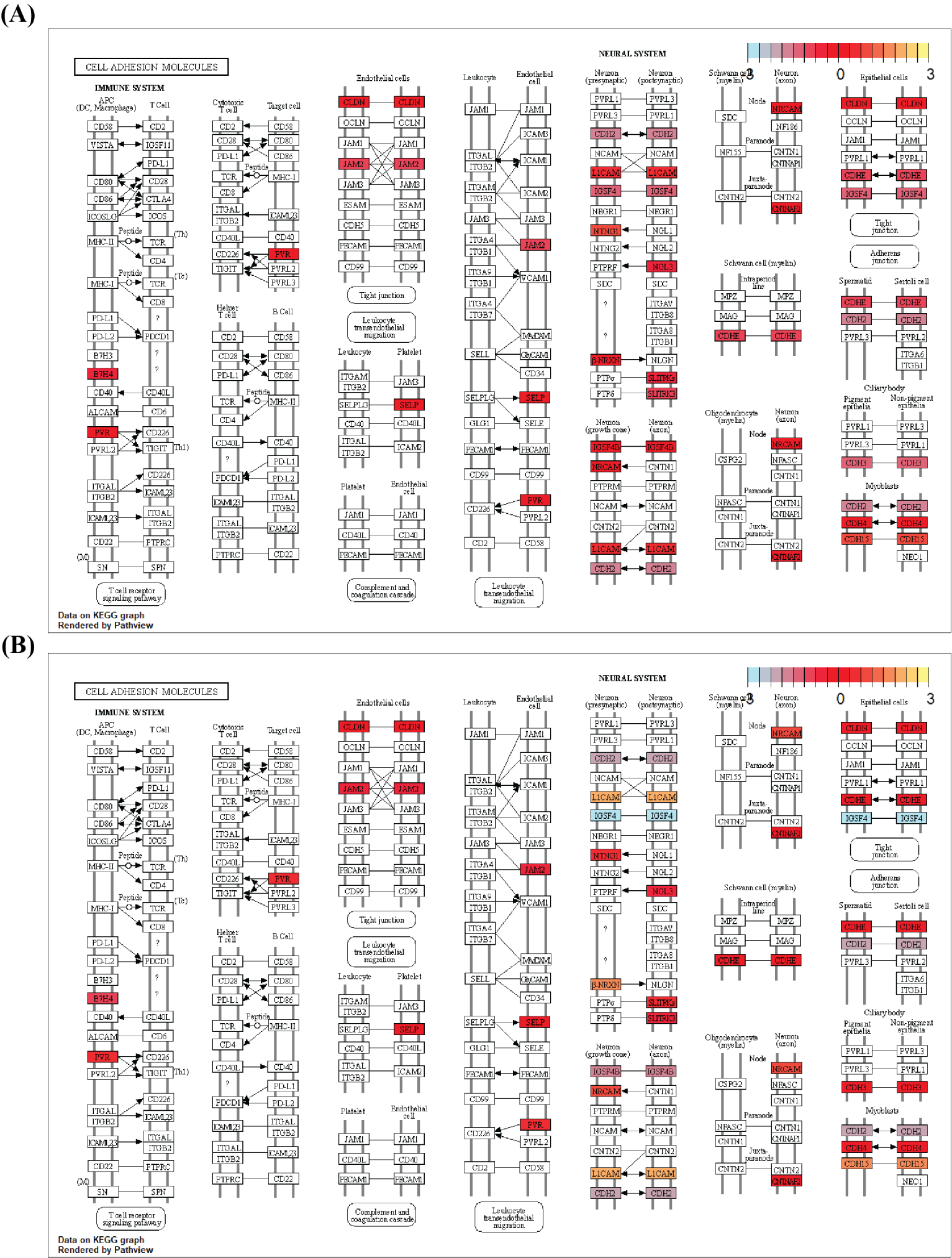

Supplement: S8 Fig — (A) APWG05 (PDX.1B). (B) V1-organoid (PDO.1). (TIF) [file pcbi.1011754.s014.tif]

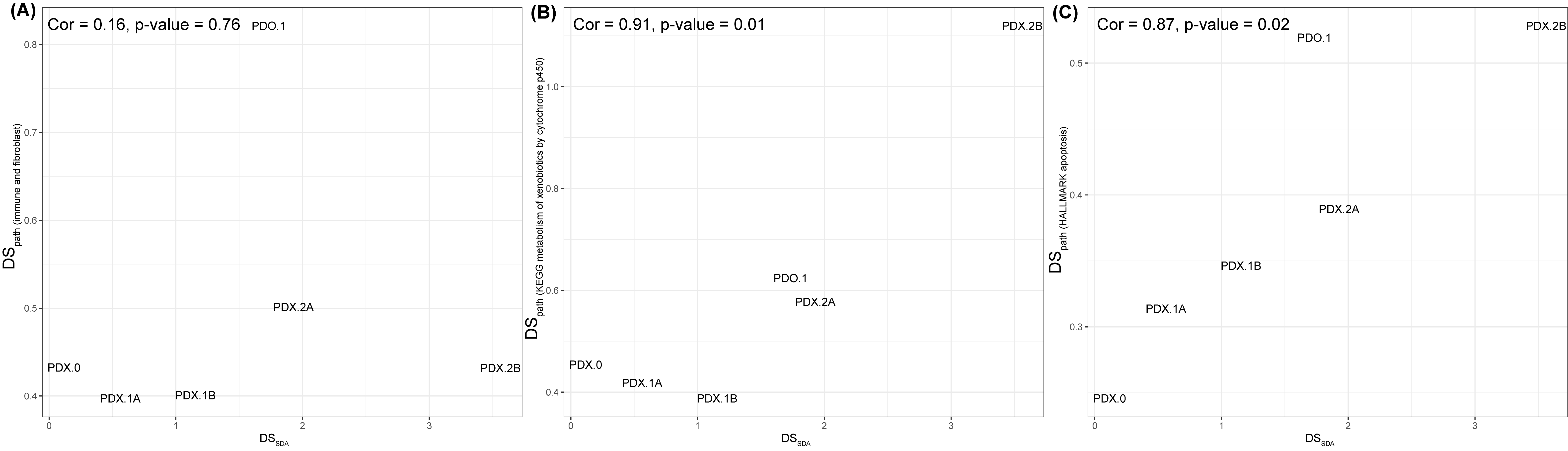

Supplement: S9 Fig — (TIF) [file pcbi.1011754.s015.tif]
