# Supplementary material for: Systems approach for congruence and selection of cancer models towards precision medicine
Source: PLoS Comput Biol. 2024 Jan 10;20(1):e1011754. doi: 10.1371/journal.pcbi.1011754 (PMC10805322; doi:10.1371/journal.pcbi.1011754)
Supplement: Attachment — Submitted filename: 231122_CASCAM_response_letter.docx [file pcbi.1011754.s016.docx]

November 21, 2023

RE:	Revisions for PCOMPBIOL-D-23-00715: “Systems approach for congruence and selection of cancer models towards precision medicine”

Dear Editor, Associate Editor, and Reviewers,

We would like to thank you for the time and effort in reviewing our manuscript. We are pleased to see the reviewers’ recognitions on the value of our study, and we highly appreciate the insightful and constructive comments to significantly improve our paper.

In this revision, we have made substantial efforts according to the critiques. Below are point-by-point responses to the reviewers' concerns. Our responses below and changes in the manuscript are highlighted in blue. We hope you’ll find the revision suitable for publication in PLOS Computational Biology.

Thank you very much for your attention and best regards!

George C. Tseng

Professor and Vice Chair for Research

Department of Biostatistics, University of Pittsburgh

Steffi Oesterreich

Professor and Shear Family Foundation Chair in Breast Cancer Research

Department of Pharmacology and Chemical Biology, University of Pittsburgh

Adrian Lee

Professor and Pittsburgh Foundation Chair in Precision Medicine

Department of Pharmacology and Chemical Biology, University of Pittsburgh

**Reviewer 1**

“Zou et al have provided a useful, clearly written manuscript describing the method CASCAM. CASCAM builds off of the expression alignment tool Celligner to include machine learning methods for cancer cell type classification with an interpretability module. These additions provide utility that will benefit the research community, and the authors have provided an accessible GitHub implementation of the software.

Overall, I have a positive assessment of the work, with two relatively minor comments.”

Response: Thanks for the positive comment.

“1. Line 306: “Fig. 6A showed a clear pattern that PDX.0 has almost perfect DSSDA congruence to represent ILC but the deviance score increased with increasing passage numbers (also see Table 2 Column 5), indicating that the xenografts may evolve and be affected by the microenvironments in mice and deviate from the original tumor over time.”

A common difference between later passage xenografts and early passages is that stromal cells such as fibroblasts and immune cells do not persist. The authors’ statement suggests, on the other hand, that they believe that the cancer cells evolve between early and late passages. The authors should clarify this point by doing a more thorough pathway analysis using their module 3 to determine whether the difference in assignment of ILC status is related to fibroblast and immune pathways.”

Response: Thank you for the insightful suggestion. We agree with this comment, ie support the notion that not only changes in the tumor cells, but evolution and loss of stroma cells contribute to increased deviance score over time. To provide data for this, we have performed additional analysis to elucidate the role of fibroblasts and immune cells in the deviance observed across PDX passages. Specifically, we queried the Molecular Signatures Database (MSigDB) using the keyword combination “fibroblast AND immune” in all the pathways and “fibroblast OR immune” in the hallmark gene sets (H) and curated gene sets (C2) (1,2). 108 pathways were identified, and 7 of them were manually selected according to their representative roles in immune and fibroblast functions. The combination of these 7 pathways contains 357 genes, of which 35 were identified as differentially expressed (Fisher exact test p-value = 0.053).

We then calculated the $DS_{path}$ (assuming the 357 genes is a pathway) for each of the six cancer models under investigation. Our analysis showed a weak positive correlation between $DS_{SDA}$and $DS_{path}$ (Pearson correlation = 0.16, p-value = 0.76; see Figure A(A) below and Supplement Figure 9 in the manuscript), suggesting that fibroblast and immune functions provide a minor contribute to the deviation in PDX models across passages. However, other pathways, such as metabolism of xenobiotics by cytochrome P450 (KEGG ID: hsa00980) and cell death (MSigDB ID: M5902), exhibited high correlation with $DS_{SDA}$ (Pearson correlation = 0.91 and 0.87, p-value = 0.01 and 0.02, respectively; see Figure A(B) and A(C) below and Supplement Figure 9 in the manuscript). These preliminary in silico analyses show a weak association between PDX deviance over passages and immune/fibroblast function. This result, however, is only suggestive due to small sample size (N=6 cancer models) and bulk RNA analysis. A solid conclusion would require higher-resolution data, such as scRNA-seq. We have added this investigation to the manuscript (quoted below).

*Figure A. Correlation between* $DS_{path}$ *and* $DS_{SDA}$ *for three pathways. (A)* *MSigDB fibroblast and immune; (B) KEGG metabolism of xenobiotics by cytochrome p450; (C) HALLMARK apoptosis*

*“Stromal cells, such as fibroblasts and immune cells, have been reported to diminish across PDX passages* (3)*. To assess their role in passage variability (Fig. 6A), we queried the Molecular Signatures Database (MSigDB) with “fibroblast AND immune” for all pathways and “fibroblast OR immune” for hallmark (H) and curated (C2) gene sets* (1,2)*. This yielded 108 pathways, of which 7 were selected for their relevance to immune and fibroblast functions, comprising 357 genes. Among these, 35 were differentially expressed (Fisher's exact test, pval = 0.053). Analysis across six cancer models showed a weak correlation between DS_SDA_ and a derived score, DS_path_, based on these 357 genes (Pearson correlation = 0.16, pval = 0.76, Fig S8A in S1 file). Conversely, pathways such as xenobiotic metabolism by cytochrome P450 (KEGG ID: hsa00980) and cell death (MSigDB ID: M5902) are strongly correlated with DS_SDA_ (Pearson correlation = 0.91 and 0.87; pval = 0.01 and 0.02, respectively, Fig S8BC in S1 file). These results seem to indicate a minor role for immune and fibroblast functions in PDX passage variability. This conclusion is, however, suggestive and should be interpreted with caution due to the small number of cancer models (N = 6) and the use of bulk RNA analysis. For a more solid conclusion, future studies should employ higher-resolution methods such as scRNA-seq.”*

“2. To clarify why the PDO is identified as ILC, it would be useful for the authors to determine from PDMR whether the PDO was derived from the ILC-classified PDX lineages, the non-ILC PDX lineages, or through some other lineage not downstream of the observed PDX models.”

Response: Thanks for the excellent suggestion. We traced back this patient (ID: 171881-019-R) in the PDMR database and did not find any histological information beyond a diagnosis of “invasive breast carcinoma”. We posit that the tumor likely originated as ILC due to ILC classification in all PDX and PDO samples annotated to this patient, and the histology was incorrectly assigned in the database. We have added this concept to the manuscript (quoted below).

*“We next investigated clinical annotation of the patient PRMR ID:171881-019-R. Although the specific histological subtype was not annotated in the database, an insertion frameshift mutation (p.T115Nfs*53) for $CDH1$ was reported. Since loss of $CDH1$ is a key determinant of ILC, it strongly suggests that the original tumor was of lobular histology. It validates the congruence analysis above that the PDO and three PDXs (PDX.0, PDX.1A and PDX.1B) are representative of the ILC tumor cohort compared to IDC.”*

“Line 389: “PDO is widely believed to be a highly conserved cancer model promising for precision medicine development and superior to PDX [7,50].”

This statement is not accurate. Although PDOs have promising uses, there is not a consensus that they are superior to PDXs. For example, PDX drug treatment experiments remain the standard used by the NCI for deciding on advancement to human clinical trials. Please adjust your description.”

Response: Thanks for pointing this out, and we agree with the reviewer’s statement. We have therefore modified our description with a more thorough view from the literature regarding the pros and cons of PDO and PDX.

*(The end of the fifth paragraph in the Discussion Section): “In addition, our result shows inconclusive comparative performance of PDO and PDX, which is consistent with current reports in the literature. PDOs offer advantages such as being an in vitro system with lower costs yet maintaining cellular heterogeneity* (4,5)*. However, they lack of immune system components and loss of stromal components over time represent limitations in representing the tumor microenvironment* (6)*. On the other hand, PDXs preserve the original tumor architecture and include stromal and at least some immune components* (7,8)*, but again, PDX development and maintenance is time and resource intensive.”*

**Reviewer 2**

“This manuscript is focused on the analysis of breast cancer cell lines, PDOs, PDXs and breast cancer samples. They have proposed a three module analysis system. Starting with the usual issue of batch correction and normalization they have integrated various datasets, demonstrating that newer methods offer advances over some well used modules. The second module uses a machine learning approach to assign samples and the third module offers information about pathways. This was applied to breast cancer cell lines and PDOs in two case studies, both being focused on ILC. The manuscript is well written, the CASCAM package is available in R and it is a valuable resource to those without established analysis pipelines in their lab.”

Response: Thank you very much for the very positive evaluation of our work.

“The major limitation is that the analysis and proof of concept work was limited to ILC samples. This (as they state) only composes 10-15% of breast cancer. As such, this does NOT adequately profile the CASCAM package for general use. In order for the manuscript to be widely read and applied, additional analysis should be completed for other major breast cancer subtypes. At a minimum I would request that HER2+ve, ER+/PR+ and TNBC experiments be completed and then added to the figures that were provided. Given that the R package is complete, this request does not seem excessive and would provide a much more interesting and important validation.”

Response: We highly appreciate the insightful critique and suggestion. Following the comment, we have conducted additional analyses including major breast cancer subtypes, HER2+, ER+/PR+, and TNBC, using TCGA and CCLE data. We employed a one-vs-rest approach, applying the same statistical thresholds as in our ILC analysis ($pval\left( DS_{SDA} \right)>0.05$ & $P_{SDA}>0.8$), to identify representative cell lines for each subtype. The result, summarized in Table A (Table S5 in the manuscript) below, demonstrated a 92.6% agreement between annotation and prediction, with discrepancies in only two cell lines (HCC1500 and UACC8122/0.074, out of a total of 27 cell lines). Disagreement may be due to the fact that cell lines can exhibit heterogeneity and plasticity under different conditions (9). For instance, HCC1500 has been annotated differently across multiple studies, ranging from ER+/HER2- to ER-/HER2- (10–14). This heterogeneity might explain the imprecise prediction of this cell line but might also point towards unique aspects of this model which will be helpful for the research community.

|  | | Prediction | | | |
| --- | --- | --- | --- | --- | --- |
|  |  | ER+/PR+/HER2- | HER2+ | TNBC | None |
| Annotation | ER+/PR+/HER2- | 5 | 0 | 0 | 1 |
|  | HER2+ | 1 (UACC812) | 8 | 0 | 3 |
|  | TNBC | 1 (HCC1500) | 0 | 12 | 12 |

Table A. Confusion matrix of genome-wide prediction

We found 12 cell lines predicted to be TNBC at the genomic level and further refined this list to identify the most representative model in terms of the WNT signaling pathway. The WNT signaling pathway plays an important role in TNBC, affecting various cellular processes such as metastasis, cell proliferation and apoptosis (15–17). HCC1599 and BT549 were found to have the best and worst congruence, respectively, with respect to this pathway. Thus, CASCAM is useful in and suitable for identifying biologically relevant cell line models outside of ILC and in other cancer types.

We have added all the details and figures into the revised manuscript, “*Case study 2: selection of cell line for TNBC”*. We believe our edits in response to this suggestion have substantially strengthened the manuscript and broadened the applicability of CASCAM.

“Other concerns are all minor, from the use of contractions in the manuscript to Figure 5 Panel C being overly pixelated.”

Response: We appreciate the reviewer's attention to detail. We have revised the manuscript to ensure the correct use of contractions (highlighted in blue), thereby adhering to academic writing standards.

Additionally, Figure 5 Panel C has been revised to focus on the epithelial cells within the KEGG cell adhesion pathway. This allows for a direct comparison between BCK5 and CAMA1 with higher figure resolution.

**Reviewer 3**

“Major comments:

1) Virtually all claims made by authors, that CASCAM is unique with regards cancer model classification and prioritization, could also be performed with currently available methods based on downstream analysis. For instance, by raking cancer models using classification scores from current methods, users could then select the models with higher classification probability and then, identify pathways and genes conserved or divergent between the ranked models and their in vivo tumor counterparts. The authors simply implemented a downstream metric based on the sparse linear discriminant analysis (SDA) projected space that would allow them to evaluate some of the cancer model heterogeneity relative to the bulk tumor measurements.

CASCAM lacks computational novelty, it simply combines already available methodologies (Cellaligner, SDA, pathway analysis) into a workflow for cancer model prioritization. In the lack of experimental follow-up and in absence of clear biological insights related to the biology of their tumor of interest, it feels like CASCAM does not represent a significant advance over prior methods on the same task, especially because there were no additional comparisons on the molecule profiles of cancer models identified by ElasticNet or 2D-Hybrid-CNN, etc.”

Response: We appreciate the reviewer’s critique to better strengthen the motivation, innovation, and necessity of CASCAM to the readership. Firstly, as we note in the original submission, the existing extensive literature (Table S1; 10+ papers) investigates the problem of cell line choice from a purely machine learning perspective with little to no systems approach to gene and pathway level congruence analysis. Although seemingly straightforward, CASCAM is the first framework, to the best of our knowledge, to streamline the analysis from data harmonization (Module 1), pre-screening by machine learning (Module 2), and mechanistic investigation (Module 3), and providing careful development and evaluation in each intermediate step. Depending on the specific cancer type and mechanism of interest, CASCAM provides multi-resolution evaluation of congruence from genome-wide (Figure 4A), to pathways (Figure 5A), to genes (Figure 5B), and to gene regulatory network (Figure 5C). In contrast, existing approaches based on pure machine learning cannot provide such systems and mechanistic investigation.

Secondly, in response to the reviewer’s concern of using classification probability from machine learning to prioritize the cancer models, we have done additional analyses to show that the classification probability is often not sufficient for cancer model selection. Of the 16 machine learning methods evaluated (Table 1 left panel in the manuscript), many methods provide hard label prediction, including ElasticNet and 2D-Hybrid-CNN noted by this reviewer, which generates no ranking. Only a few can provide soft predictions with classification probabilities (or classification scores). Figure B below (Fig S1 in the manuscript) shows classification probabilities using LDA, SDA and RSLDA in the ILC case study, where many samples classified to ILC have classification probability very close or equal to one. The result shows the inability of genome-wide machine learning alone (using classification probability) to prioritize to cancer models in general. This motivates the strategy of combining classification probability and deviance score (Figure 4A) to prioritize and pre-screen cancer models in Module 2.

*Figure B. Assignment probabilities of three different methods. (A) LDA; (B) SDA; (C) RSLDA*

Thirdly, although SDA as well as several other machine learning methods provide explicit or implicit gene selection, the selected genes are meant to construct a prediction model that achieves high generalization prediction accuracy. Genes selected in machine learning can miss critical driver or regulatory genes and are not suitable for mechanistic understanding (18). As a result, machine learning (by SDA in CASCAM) is only used for pre-screening cancer models in Module 2. The gene-based and pathway-based deviance score and mechanistic investigation in Module 3 are critical for biologically insightful congruence analysis.

Lastly, as suggested by this reviewer’s nice subsequent critique (Critique 3 below), we have performed specificity (negative control) evaluation using 24 liver cancer cell lines in the Case Study 1 ILC analysis. The result further verifies the shortcoming of machine learning approach alone (Module 2) and the necessity of mechanistic investigation (Module 3) when irrelevant cell lines belonging neither to ILC nor IDC are included so the specificity can be guaranteed (see the new section “Specificity evaluation for irrelevant cell lines from other cancers”).

In summary, we show that machine learning alone does not provide an adequate answer to cancer model congruent analysis and selection. The purpose of CASCAM is not to propose another innovative machine learning method. Instead, CASCAM is a thoroughly evaluated systems approach for evaluating congruence of cancer models in multiple resolutions (genome-wide, pathway, gene and gene regulatory network).

In this revision, we have strengthened the rationale and innovation of CASCAM in the introduction, result and method sections, highlighted in the manuscript. Once again, we sincerely thank this reviewer for their constructive critique to improve our paper. Below are selected quotes in this revision.

*(Author’s summary): “Existing methods are machine learning based and are limited to prediction using genome-wide information without mechanistic insights. To address this, we developed a comprehensive suite of bioinformatics tools, namely Congruence Analysis and Selection of CAncer Models (CASCAM). The framework develops a multi-stage systems approach to quantify pathway and gene specific congruence and allow prioritization and selection of the most congruent cancer model(s), which provides a paradigm shift towards gene regulatory and systems investigations.”*

*(The third paragraph in ‘Transparent machine learning pre-selection’): “Many methods, including ElasticNet and 2D-Hybrid-CNN, produce hard label predictions, while only a few, such as LDA, SDA, and RSLDA, offer soft assignments with classification probabilities. However, these soft assignment probabilities often tend towards binary values, as evidenced in Fig S1 in S1 file. This limits their utility for model prioritization and underscores the limitation of relying solely on classification probabilities for model prioritization. Consequently, we devised a strategy that combines classification probabilities with deviance scores to more effectively prioritize and pre-screen cancer models, as detailed in Module 2.”*

*(The first paragraph in ‘Pathway and mechanistic-based selection of cancer model(s)’): “While SDA and other machine learning methods offer gene selection aimed at maximizing predictive accuracy, they can miss driver or regulatory genes essential for mechanistic understanding. Therefore, in our CASCAM framework, machine learning via SDA serves only as a pre-screening tool in Module 2. The subsequent gene-based and pathway-based deviance scores, along with mechanistic investigations in Module 3, are crucial for conducting biologically insightful congruence analysis.”*

“2) The authors also claim that their machine learning model, based on SDA provides an interpretable machine-learning model because gene selection leads to gene signatures that make the model interpretable. This is not entirely true as far as I can tell, the model itself does not provide information for which features/genes are actually important to drive the classification scores. Several machine learning methods use gene signatures as features for training. This does not make such models fully interpretable.”

Response: We thank the reviewer's concern regarding “interpretable” machine learning. In the first submission, we called SDA in CASCAM interpretable for two main reasons: 1) SDA performs gene selection, thereby identifying contributing genes; 2) SDA assigns weights to each selected gene that linearly project gene expression data in the direction that maximally differentiates between IDC and ILC. These weights serve as gene importance scores, rendering model interpretation.

Indeed, different researchers may have different criteria for calling a machine learning model “interpretable”. To mitigate any potential confusion or debate, we have changed the description to “*transparent* machine learning” (19,20) in the revised manuscript, which is loosely in contrast to “*black-box*” machine learning methods. However, since machine learning by SDA in CASCAM only serves as a pre-screening tool in Module 2 and the selected genes are not used for mechanistic investigation, we do not overly emphasize the transparent machine learning property in CASCAM.

“3) Authors should also include a comparison of cancer models other than their cancer of interest, to determine whether the pathway and gene-level modules of CASCAM indeed capture biological processes specific to cancer models for ILC.”

Response: We sincerely appreciate this excellent suggestion. In this revision, and in response to previous reviewer’s comments we have shown that CASCAM can identify relevant cell line models for other subtypes of breast cancer such as HER2 and TNBC. Further, as a negative control, we have selected 24 liver cancer cell lines from CCLE to the ILC case study to assess the specificity of our CASCAM model. In Figure C(A) below (Fig S3A in the manuscript), 9 of the 24 cell lines are predicted to ILC and 10 are predicted to IDC by SDA. Combined with deviance score criteria, five of the 6 cell lines predicted to ILC are selected for Module 3 analysis. As shown in Figure C(B) below (Fig S3B in the manuscript), all five liver cell lines have extremely large $DS_{path}$ scores in all selected pathways, showing their deviance from ILC. The result nicely shows the shortcoming of machine learning pre-screening in Module 2 when a cell line belongs to neither of ILC or IDC while mechanistic investigation in Module 3 would expose it to increase specificity. In this revision, we have included these findings in the subsection *Case study 1: selection of cell line for ILC*.

*(new paragraph ‘Specificity evaluation for irrelevant cell lines from other cancers’): “To evaluate the specificity of CASCAM when an irrelevant cell line belonging to neither ILC nor IDC is included, we selected 24 liver cancer cell lines from CCLE as negative controls in the analysis. As shown in Fig S3A, five of the 24 liver cell lines were selected under the same classification probability* $P_{SDA}$ *and deviance score* $DS_{SDA}$ *criteria. When further analyzed by the deviance score heatmap of selected pathways in Fig S3B, the five selected liver cell lines exhibit extremely large deviance scores in all pathways, demonstrating the increased specificity to avoid false positives by pathway-based mechanistic evaluation in Module 3 and the shortcoming of relying on Module 2 machine learning alone.”*

Figure C. Cross-cancer validation of CASCAM

Minor comments:
1) Authors should cite Figure panels, not the approaches themselves (A-D), as they do in Figure 1.

Response: Thanks for pointing this out. We have revised the manuscript to ensure that figure panels are cited.

*(the first paragraph in “Data harmonization between cancer model and tumor transcriptomic data”): “Biased separation of tumors and cell lines was clearly found when no normalization or conventional quantile normalization were implemented (see Fig. 2A and B). Combat and Celligner using BC tumors and BC cell lines (see Fig. 2C and D) produced improved normalization although systematic bias was still observed from small clusters of cell lines, showing insufficient quality of data harmonization. In contrast, Celligner using pan-cancer tumors and pan-cancer cell lines (see Fig. 2E) best eliminated batch effects between BC tumors and BC cell lines”*

2) It is not clear the rationale by which authors select pathways based on large (and not small) P-values. In my experience this is counterintuitive as authors seem to select cell lines that are not statistically significant. I understand what they mean, but this should be more clearly stated to avoid confusion. See lines 165-166, for instance.

Response: Thanks for the comment. To address this, we have inserted a clarification sentence in the fourth paragraph in “Transparent machine learning pre-selection”: “*We note that the selection of cell lines with large p-value is not conventional since hypothesis testing with small p-value is usually designed for capturing differences, while we are interested in congruence (i.e., no difference) here.”*

3) Figure 5C does not add anything to the manuscript. The resolution is also pretty bad.

Response: Thank you for your comment. We have redesigned Figure 5C to compare CAMA1 (high congruence) and BCK4 (low congruence) in the epithelial cell part of the KEGG cell adhesion pathway. This provides a more visually intuitive pathway comparison with higher resolution.

4) Authors should more clearly state their conclusions and better explain their results. In line 330, for instance, the authors describe the violin plot but an actual conclusion is not clearly stated.

Response: Thanks for pointing out. We revised the sentence to more explicitly state the conclusion drawn from the violin plot. Additional modifications have been made throughout the manuscript to ensure that our conclusions are clearly articulated and that our results are comprehensively explained. Below are selected quotes in this revision.

*(the last paragraph in “Transparent machine learning pre-selection”): In Fig 4B, the 9 unbiased-selected and 1 manually-included cell lines were ranked by* $DS_{SDA}$ *with 95% confidence interval provided, with SUM44PE and HCC2185 occupying the highest and lowest ranks, respectively.*

*(the fifth paragraph in Case Study 3): “The comparison between PDX.1B and PDO in violin plots, with the gene expression distribution in IDC and ILC tumors as reference, verified that PDX.1B was a better model for ILC Fig 6C and Fig S7 in S1_file).”*

**Reference**

1.	Liberzon A, Birger C, Thorvaldsdóttir H, Ghandi M, Mesirov JP, Tamayo P. The Molecular Signatures Database (MSigDB) hallmark gene set collection. Cell Syst. 2015 Dec 23;1(6):417–25.

2.	Kanehisa M, Goto S. KEGG: kyoto encyclopedia of genes and genomes. Nucleic Acids Res. 2000 Jan 1;28(1):27–30.

3.	Borodovsky A, McQuiston TJ, Stetson D, Ahmed A, Whitston D, Zhang J, et al. Generation of stable PDX derived cell lines using conditional reprogramming. Mol Cancer. 2017 Dec 6;16(1):177.

4.	Li Y, Gao X, Ni C, Zhao B, Cheng X. The application of patient-derived organoid in the research of lung cancer. Cell Oncol . 2023 Jun;46(3):503–19.

5.	Spagnol G, Sensi F, De Tommasi O, Marchetti M, Bonaldo G, Xhindoli L, et al. Patient derived organoids (PDOs), extracellular matrix (ECM), tumor microenvironment (TME) and drug screening: state of the art and clinical implications of ovarian cancer organoids in the era of precision medicine. Cancers [Internet]. 2023 Mar 30;15(7). Available from: http://dx.doi.org/10.3390/cancers15072059

6.	Zhang W, Zheng X. Patient-derived xenografts or organoids in the discovery of traditional and self-assembled drug for tumor immunotherapy. Front Oncol. 2023 Apr 4;13:1122322.

7.	Pu X, Zhang R, Wang L, Chen Y, Xu Y, Pataer A, et al. Patient-derived tumor immune microenvironments in patient-derived xenografts of lung cancer. J Transl Med. 2018 Nov 26;16(1):328.

8.	Liu W, Cui Y, Zheng X, Yu K, Sun G. Application status and future prospects of the PDX model in lung cancer. Front Oncol. 2023 Mar 24;13:1098581.

9.	Dai X, Cheng H, Bai Z, Li J. Breast cancer cell line classification and its relevance with breast tumor subtyping. J Cancer. 2017 Sep 12;8(16):3131–41.

10.	Riaz M, van Jaarsveld MTM, Hollestelle A, Prager-van der Smissen WJC, Heine AAJ, Boersma AWM, et al. miRNA expression profiling of 51 human breast cancer cell lines reveals subtype and driver mutation-specific miRNAs. Breast Cancer Res. 2013 Apr 19;15(2):R33.

11.	Kao J, Salari K, Bocanegra M, Choi Y-L, Girard L, Gandhi J, et al. Molecular profiling of breast cancer cell lines defines relevant tumor models and provides a resource for cancer gene discovery. PLoS One. 2009 Jul 3;4(7):e6146.

12.	Gazdar AF, Kurvari V, Virmani A, Gollahon L, Sakaguchi M, Westerfield M, et al. Characterization of paired tumor and non-tumor cell lines established from patients with breast cancer. Int J Cancer. 1998 Dec 9;78(6):766–74.

13.	Neve RM, Chin K, Fridlyand J, Yeh J, Baehner FL, Fevr T, et al. A collection of breast cancer cell lines for the study of functionally distinct cancer subtypes. Cancer Cell. 2006 Dec;10(6):515–27.

14.	Jiang G, Zhang S, Yazdanparast A, Li M, Pawar AV, Liu Y, et al. Comprehensive comparison of molecular portraits between cell lines and tumors in breast cancer. BMC Genomics. 2016 Aug 22;17 Suppl 7(Suppl 7):525.

15.	Merikhian P, Eisavand MR, Farahmand L. Triple-negative breast cancer: understanding Wnt signaling in drug resistance. Cancer Cell Int. 2021 Aug 10;21(1):419.

16.	Pohl S-G, Brook N, Agostino M, Arfuso F, Kumar AP, Dharmarajan A. Wnt signaling in triple-negative breast cancer. Oncogenesis. 2017 Apr 3;6(4):e310.

17.	Bilir B, Kucuk O, Moreno CS. Wnt signaling blockage inhibits cell proliferation and migration, and induces apoptosis in triple-negative breast cancer cells. J Transl Med. 2013 Nov 4;11:280.

18.	Saeys Y, Inza I, Larrañaga P. A review of feature selection techniques in bioinformatics. Bioinformatics. 2007 Oct 1;23(19):2507–17.

19.	Diaz Ochoa JG, Marquardt A. Editorial: Transparent machine learning in bio-medicine. Front Bioinform. 2023 Aug 4;3:1264803.

20.	Zhou Y, Kantarcioglu M. On transparency of machine learning models: A position paper [Internet]. [cited 2023 Oct 10]. Available from: https://crcs.seas.harvard.edu/sites/projects.iq.harvard.edu/files/crcs/files/ai4sg_2020_paper_62.pdf
